# Revolutionizing Neurosurgery: The Cutting-Edge Era of Digitally Fabricated Cranial Stents

**DOI:** 10.7759/cureus.53482

**Published:** 2024-02-03

**Authors:** Arushi Beri, Sweta G Pisulkar, Sandeep Iratwar, Akansha Bansod, Ritul Jain, Akshay Shrivastava

**Affiliations:** 1 Prosthodontics, Sharad Pawar Dental College and Hospital, Datta Meghe Institute of Higher Education and Research, Wardha, IND; 2 Prosthodontics and Crown and Bridge, Sharad Pawar Dental College and Hospital, Datta Meghe Institute of Higher Education and Research, Wardha, IND; 3 Neurosurgery, Jawaharlal Nehru Medical College, Datta Meghe Institute of Higher Education and Research, Wardha, IND; 4 Orthodontics and Dentofacial Orthopedics, Kalinga Institute of Dental Sciences, Orissa, IND

**Keywords:** ct scan, cranioplasty, digitalisation, rehabilitation, cranial reconstruction, 3d printing

## Abstract

Cranial defects are broadly classified as either congenital or acquired. The prevalence of cranial injuries has notably increased, propelled by a heightened emphasis on aesthetics and the demand for skull reconstruction in contemporary society. Consequently, rehabilitation for these defects has also surged. Surgical correction or repair, known as cranioplasty, not only aims at aesthetic rehabilitation but also addresses psychological issues, improving social acceptance and overall performance. Amid evolving trends, the availability of advanced biomedical tools, technologies, and materials empowers surgeons and prosthodontists, leading to improved outcomes in aesthetics and functionality. One noteworthy technique highlighted in this case report involves using bone cement in conjunction with polymethyl methacrylate, adding novelty to the approach. The interdisciplinary management team, consisting of prosthodontists and neurosurgeons, played a pivotal role in improving neurological status and cosmetic outcomes for the patients.

## Introduction

There are two types of craniofacial defects: congenital and acquired. The incidence of surgical correction of cranial injuries has significantly increased due to the public's increased awareness of aesthetics and the growing need for skull reconstruction in the contemporary day [1]. Consequently, the rehabilitation of these defects has experienced a notable surge in recent times. The surgical correction or repair of cranial defects, known as cranioplasty, seeks to achieve more than just aesthetic rehabilitation. It addresses underlying psychological issues, fostering not only relief but also an improvement in social acceptance and overall performance for the affected individuals. In response to the dynamic trends in the medical field, there has been a considerable evolution in biomedical tools, technologies, and materials. These advancements have provided surgeons and prosthodontists with an expanded array of resources, facilitating improved outcomes in functionality and aesthetics. This article aims to present case reports, focusing on the restoration of cranial defects while concurrently bolstering the psychological confidence of the patients. One noteworthy technique emphasized in this case series involves the innovative use of bone cement in combination with polymethyl methacrylate (PMMA), adding a novel dimension to the overall approach. The interdisciplinary nature of the management team, consisting of prosthodontists and neurosurgeons, played a pivotal role in achieving holistic improvements for the patients. This collaborative approach not only addressed the aesthetic concerns but also contributed significantly to enhancing neurological status and overall cosmetics [2]. The multifaceted benefits of cranioplasty extend beyond mere cosmetic rehabilitation. While the protective and aesthetic aspects are crucial, the procedure significantly contributes to the patient's overall psychological well-being. The relief provided by cranioplasty is not limited to the physical reconstruction of the skull; it extends to the alleviation of psychological challenges, ultimately leading to improved social integration and overall performance. These innovations empower healthcare professionals to tailor interventions more precisely, not only ensuring the restoration of physical form but also addressing the intricate psychological aspects associated with cranial defects [3-5].

The evolving landscape of modern dentistry has given rise to advancements in the realm of tissue regeneration, commonly referred to as “tissue engineering.” This innovative field integrates molecular biology concepts and techniques, particularly focusing on stem cell implantation followed by targeted differentiation to achieve specific functions. In the context of maxillofacial structures, where aesthetic considerations are paramount, tissue engineering holds significant promise.

Key players in the process of tissue regeneration are bone morphogenic proteins from the transforming growth factor-β family, along with various polypeptide growth factors. These elements play a central role in fracture healing, contributing to the restoration of both form and function. Maxillofacial structures, being fundamental components of aesthetics, often require reconstruction when defects are present. The assessment of patient not only aids in effective communication between healthcare professionals and patients but also aligns with the broader goal of fostering an empathetic approach in medical care. In essence, the integration of tissue engineering principles into maxillofacial reconstruction signifies a transformative approach to addressing both the physiological and psychological aspects of patients. By focusing not only on the restoration of anatomical structures but also on the enhancement of overall well-being and quality of life, modern dentistry embraces a holistic perspective that aligns with the goals of patient-centered and empathetic healthcare practices.

This article sheds light on case reports that showcase the restoration of cranial defects and the simultaneous enhancement of psychological confidence in affected patients. The use of bone cement in conjunction with PMMA introduces a novel dimension to the field, underlining the importance of innovative approaches in achieving optimal outcomes. The collaborative efforts of prosthodontists and neurosurgeons, leveraging advanced biomedical tools and technologies, exemplify the strides being made in cranial rehabilitation, emphasizing the significance of a holistic and patient-centric approach.

## Case presentation

**Figure 1:**
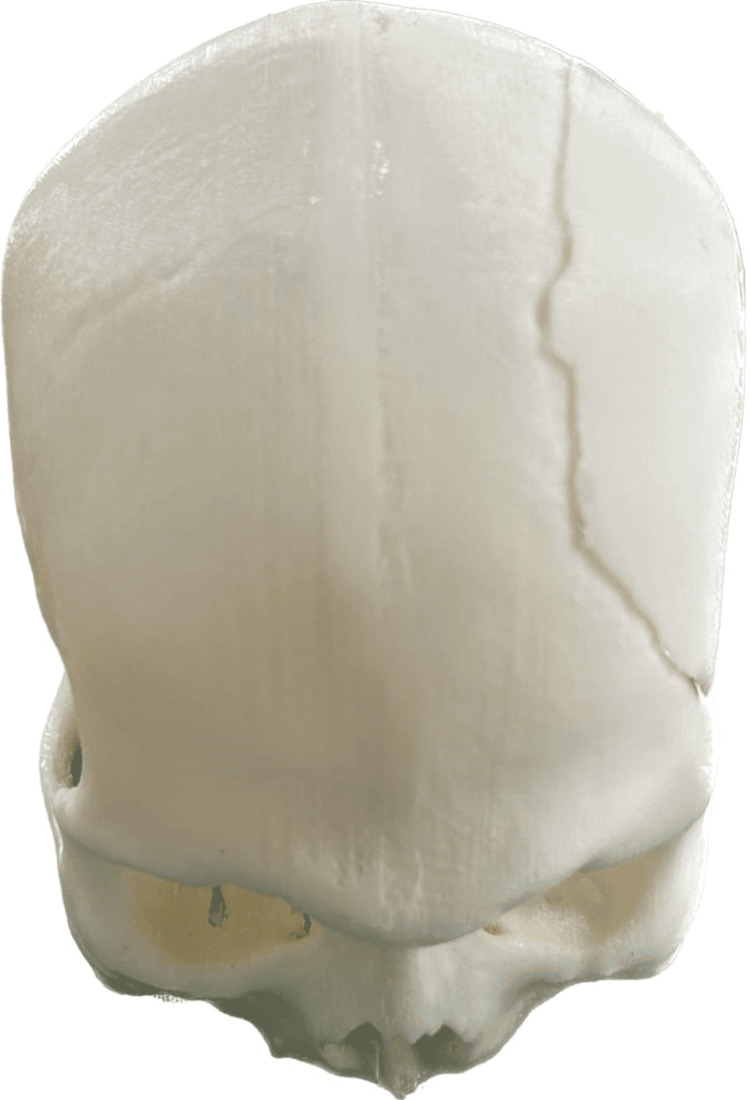
A 3D-printed model of the patient with cranial defect

This case report details the comprehensive management of a 57-year-old male patient who experienced significant cranial trauma resulting from a road accident. Subsequent surgical interventions were undertaken to address multiple bone fractures, including those affecting the head. Subsequently, the patient was referred to the Prosthodontics Department, where he presented with a chief complaint related to a postoperative calvarial defect. The procedure unfolded in two distinct phases: the prosthetic and surgical phases. In the prosthetic phase, a meticulous assessment of the calvarial defect was conducted, taking into account both the dimensions and the specific anatomical areas affected. Impressions of the defect site were then obtained, forming the basis for the creation of a detailed model. Leveraging cutting-edge digital technologies, a virtual design of the cranial prosthesis using the CT scan was meticulously crafted, ensuring a bespoke fit that would seamlessly integrate with the patient's anatomy, and a three-dimensional (3D) printed model was obtained (Figure 1).

**Figure 2:**
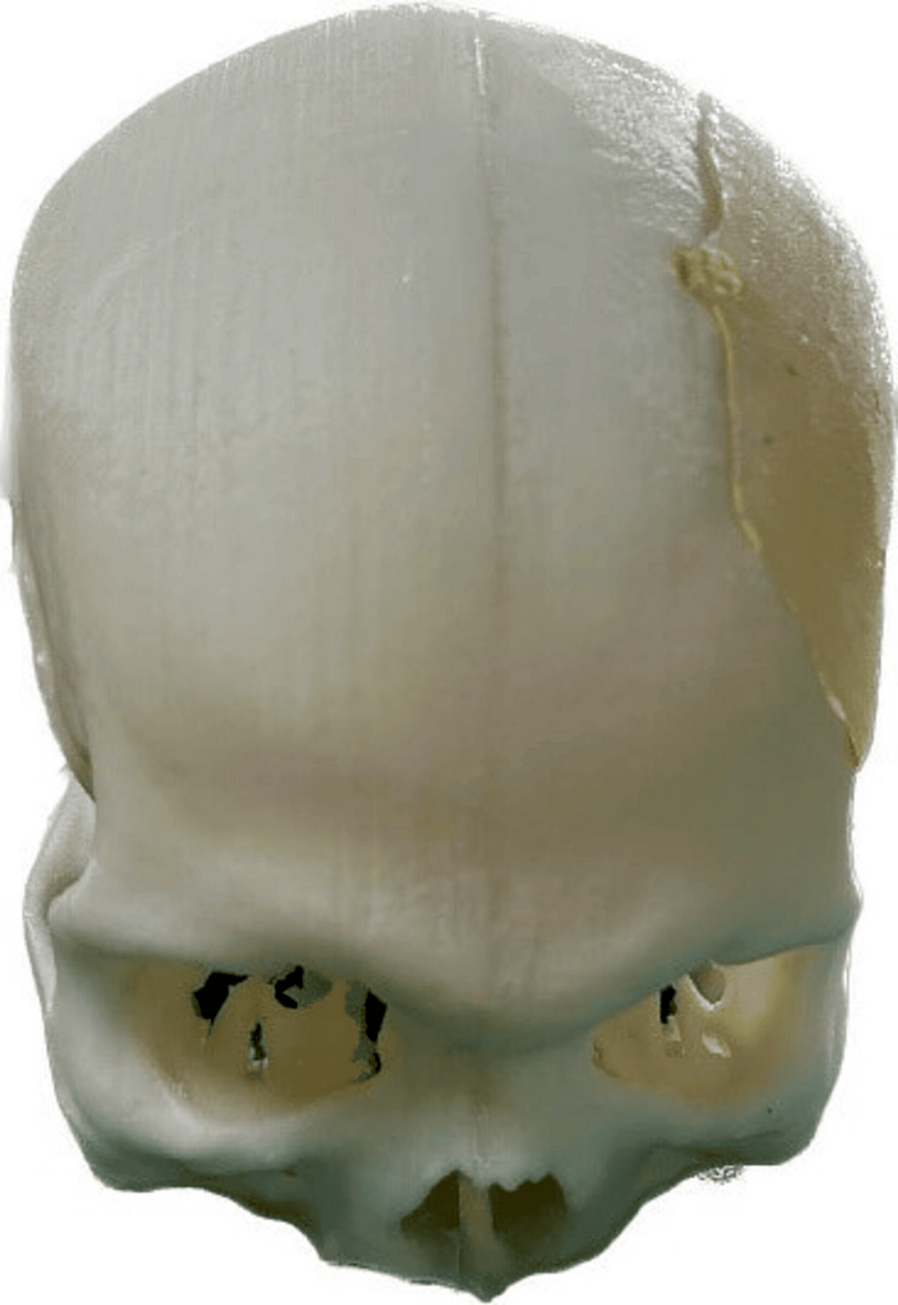
PMMA prosthesis placed on the 3D printed model PMMA, polymethyl methacrylate

The selection of high-quality materials, such as PMMA, considering both biocompatibility and durability, was a critical aspect of prosthetic fabrication to achieve not only structural integrity but also a natural appearance. Figure 2 shows the PMMA prosthesis placed on the 3D printed model.

**Figure 3:**
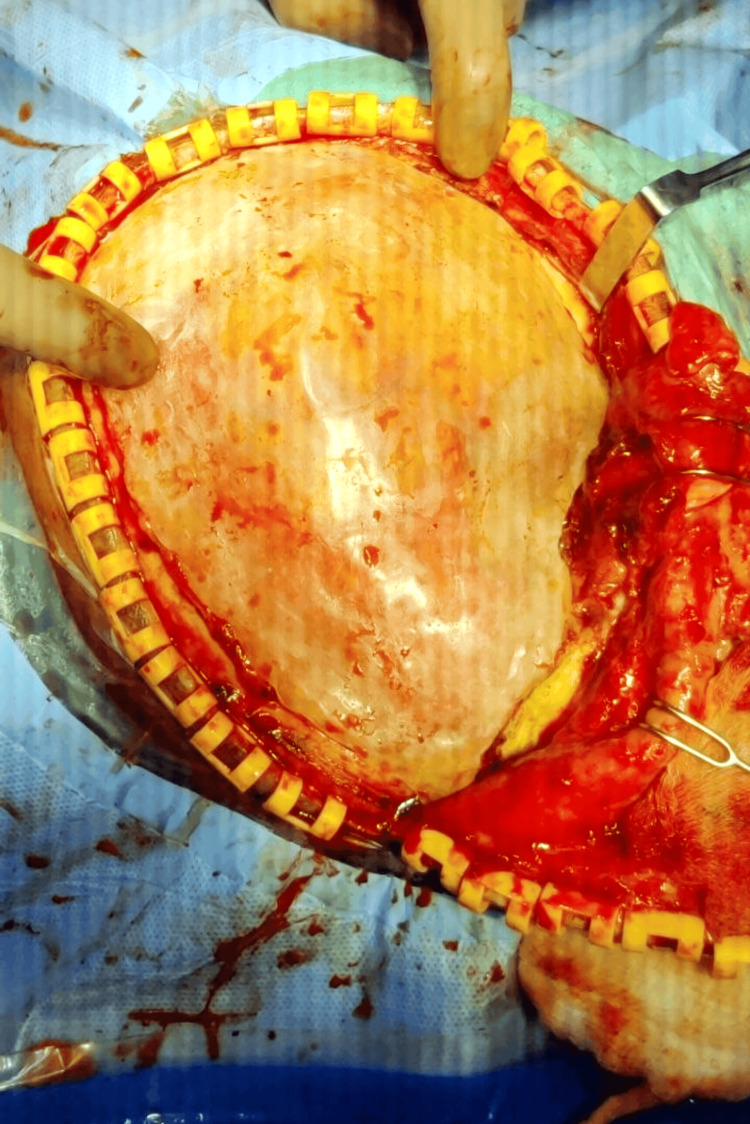
Prosthesis in defect site at the time of surgery

Transitioning into the surgical phase, a thorough preoperative assessment evaluated the patient’s overall health and readiness for the implantation of the cranial prosthesis. The carefully crafted prosthesis was then surgically implanted over the defect site, with a keen focus on achieving a secure fit and optimal alignment (Figure 3).

**Figure 4:**
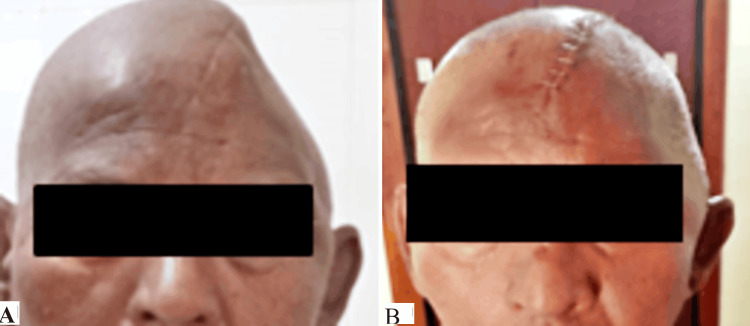
Comparison of (A) pre- and (B) post-operative images

A subsequent seven-day follow-up ensured proper integration and patient comfort. The patient underwent regular follow-up assessments, culminating in a comprehensive evaluation six months postoperatively. Notably, the absence of complications during this period underscored the success of the interdisciplinary approach, emphasizing the importance of precision in both virtual design and implantation. This case report highlights the potential for advanced prosthetic and surgical techniques to synergize, offering a personalized and effective solution for patients with extensive cranial defects, ultimately leading to successful rehabilitation and improved long-term outcomes. Pre- and post-operative photos were compared, as shown in Figure 4.

## Discussion

Cranial decompression stands as the foremost treatment for elevated intracranial pressure resulting from traumatic injuries, cerebral infarcts or hemorrhage, and tumors, among other conditions. Beyond the aesthetic considerations, the restoration of cranial defects plays a crucial role in stabilizing hemodynamic functions, neurological status, and the psychosocial well-being of patients. A myriad of rehabilitating materials are available, encompassing both metal and non-metal options, such as resin-based, ceramics, and other noble metals. Notably, titanium and PMMA are often preferred due to their biocompatibility and resilience. PMMA, a polymerized ester of acrylic acid, has been used in cranioplasty since 1940, with its application evolving over time to address various complexities [6-9].

While direct application of auto-polymerizing acrylic resin poses complications due to exothermic polymerization reactions, prefabricated prostheses, preferably heat-cured, are deemed more desirable. PMMA demonstrates excellent cosmetic results, particularly in terms of contour effects, making it a preferred choice over other materials. Reinforcing PMMA with materials such as hydroxyapatite or bone cement has been explored to enhance its durability and provide better osseo-integrative results [10-14].

Hydroxyapatite, a natural compound found in human teeth and bones, has been artificially produced and used in cranial reconstruction. Its advantages include avoiding exothermic polymerization reactions and intra-operative application on the defect site after prosthesis stabilization [15,16]. Despite its positive aspects, hydroxyapatites are prone to breakage. Combining them with titanium mesh or plates has been explored to improve durability and osseo-integrative results. Bone cement, a two-component system, has been employed in cranioplasty for its molding properties. However, its quick setting time and exothermic reaction necessitate careful manipulation to minimize adverse effects. The layer of bone cement applied intra-operatively should not exceed 5mm to avoid complications. Titanium, being radiolucent, non-ferromagnetic, and non-paramagnetic, is an attractive option. It boasts a low corrosion rate, low density, and modulus of elasticity comparable to bone. Using digital 3D printing, titanium plates can be fabricated to precisely fit the patient's anatomy. The polished dorsal surface ensures proper fit and acceptability. This comprehensive overview highlights the diverse materials and techniques employed in cranial prosthetics, each with its unique advantages and considerations [17-20].

## Conclusions

In conclusion, the use of a PMMA flap fabricated through 3D printing for cranial defect reconstruction or cranioplasty represents a promising and innovative approach. This case report highlights the potential benefits of personalized and precise implant design, allowing for optimal anatomical fit and aesthetic outcomes. The use of 3D printing technology enables customization to individual patient needs, facilitating a more accurate reconstruction of the cranial defect.

Furthermore, the reported case suggests that PMMA, known for its biocompatibility and stability, serves as a suitable material for cranioplasty, ensuring long-term durability and minimizing the risk of complications. The integration of 3D printing not only enhances surgical planning but also streamlines the manufacturing process, leading to efficient and timely reconstruction.

However, ongoing research and long-term follow-up studies are essential to validate the durability, safety, and efficacy of PMMA 3D-printed flaps in cranial defect reconstruction. As technology continues to advance, incorporating innovative materials and refining techniques, the field of cranioplasty stands to benefit from further improvements in patient outcomes and quality of life. Overall, this case report contributes valuable insights into the evolving landscape of cranial defect reconstruction, emphasizing the potential of 3D-printed PMMA flaps as a promising solution in modern neurosurgery.
